# Rugged landscapes: complexity and implementation science

**DOI:** 10.1186/s13012-020-01028-5

**Published:** 2020-09-29

**Authors:** Joseph T. Ornstein, Ross A. Hammond, Margaret Padek, Stephanie Mazzucca, Ross C. Brownson

**Affiliations:** 1 Brown School, Washington University in St. Louis, Brookings Drive, St. Louis, MO USA; 2 Department of Political Science, School of Public and International Affairs, University of Georgia, Jackson St, Athens, GA USA; 3 Center on Social Dynamics and Policy, The Brookings Institution, Massachusetts Ave, Washington DC, USA; 4 Implementation Science Center for Cancer Control and Prevention Research Center, Brown School, Washington University in St. Louis, One Brookings Drive, Campus Box 1196, St. Louis, 63130 Missouri USA; 5 Department of Surgery (Division of Public Health Sciences), Washington University School of Medicine, Washington University in St. Louis, St. Louis, 63110 Missouri USA

**Keywords:** Complexity, Agent-based modeling, Evidence-based decision-making, Mis-implementation

## Abstract

**Background:**

Mis-implementation—defined as failure to successfully implement and continue evidence-based programs—is widespread in public health practice. Yet the causes of this phenomenon are poorly understood.

**Methods:**

We develop an agent-based computational model to explore how complexity hinders effective implementation. The model is adapted from the evolutionary biology literature and incorporates three distinct complexities faced in public health practice: dimensionality, ruggedness, and context-specificity. Agents in the model attempt to solve problems using one of three approaches—Plan-Do-Study-Act (PDSA), evidence-based interventions (EBIs), and evidence-based decision-making (EBDM).

**Results:**

The model demonstrates that the most effective approach to implementation and quality improvement depends on the underlying nature of the problem. Rugged problems are best approached with a combination of PDSA and EBI. Context-specific problems are best approached with EBDM.

**Conclusions:**

The model’s results emphasize the importance of adapting one’s approach to the characteristics of the problem at hand. Evidence-based decision-making (EBDM), which combines evidence from multiple independent sources with on-the-ground local knowledge, is a particularly potent strategy for implementation and quality improvement.


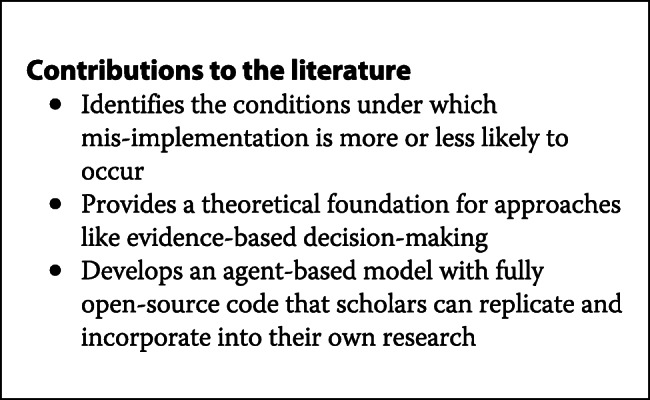


## Background

Mis-implementation is an emerging area of interest for public health researchers and practitioners [1–3]. The term refers to the premature termination of evidence-based programs or the failure to de-implement non-evidence-based programs [1], and recent evidence suggests that it is a widespread problem in public health practice. Only 58 to 62% of public health programs are evidence-based [4, 5], and 37% of chronic disease prevention staff in state health departments report discontinuing evidence-based programs [3]. While the field of implementation science has explored de-implementation of unnecessary, low-value, or overused care [6–9], there has been little study of the processes that sustain non-evidence-based programs [3, 7]. One such example is the widespread continuation of the DARE (Drug Abuse Resistance Education) program, despite many evaluations demonstrating its limited effectiveness to lower drug and alcohol use by adolescents [10, 11].

The causes of mis-implementation are not well understood, but one driver could be the sheer *complexity* of the problems that public health practitioners face. In this paper, we develop an agent-based computational model (ABM) to help understand how three types of complexity—dimensionality, ruggedness, and context-specifity—are likely to hinder the implementation of effective programs and the termination of ineffective programs. As the model makes clear, each type of complexity is best approached by a different implementation procedure.

### The three types of complexity

The first type of complexity that public health practitioners face is the need to make multiple, coordinated decisions. In the computational model, we refer to this as the *dimensionality* of a problem. Low dimensional problems require a small number of decisions (e.g., whether or not to administer a drug). These types of problems are well suited to randomized control trials (RCT), which can convincingly demonstrate the efficacy of treatment and determine the best program to implement. However, most public health programs are high dimensional, consisting of a large number of decisions and components. Many of the most pressing public health challenges involve multiple coordinated actions across multiple stakeholders, engaging with both conventional health drivers and social determinants of health [12–14]. For such multifaceted problems, RCTs are often infeasible or unethical.

The second type of complexity is called *ruggedness* (for reasons the model makes clear). Rugged problems are characterized by interdependence: the effectiveness of any one decision cannot be determined in isolation, because it depends on what other decisions are made. Consider, for example, an intervention that aims to improve rural nutrition by providing egg-laying hens to low-income families. Such a program is unlikely to be effective unless a number of complementary decisions are made, like providing veterinary vaccinations for Newcastle disease, training recipients on how to provide shelter for their hens from predators, and adequate farming assistance to produce grain crops for the hens to eat [15, 16]. The interactive effect of these many decisions may explain why research has failed to find a significant effect of egg interventions in the past.

The final type of complexity is *context-specificity*, the tendency for a program’s effectiveness to vary based on context [17]. Programs that are successful in some region or population may perform poorly when adopted elsewhere. For example, a recent study of retail-oriented tobacco control policies found the best policy combinations to be heavily dependent on the economic and spatial context [18, 19]. Similarly, whole-of-community obesity prevention interventions are well supported by evidence and can be highly effective [13, 20, 21] but successful implementation of these complex interventions often depends on careful tailoring to context [22–24]—what works in one community may not transfer to a quite different setting without local adaptation.

In the sections that follow, we develop an agent-based computational model (ABM), which represents mathematically the types of complex problems described above. As the model will demonstrate, each type of complexity is best approached by a particular set of strategies. We conclude the paper with a discussion of how public health agencies, funders, and researchers can best implement effective programs when faced with these sorts of complex problems.

## Methods

The model we develop here is a variant of the NK model—a classic in the field of evolutionary biology [25, 26]—which conceptualizes complex problem-solving as a search for high-value peaks on a *rugged fitness landscape*. This approach has provided insights in a wide range of fields, although its application to organizational decision-making in public health is novel. In theoretical biology, the rugged landscape model has been used to help understand the evolution of complex organisms [27] and the immune response [26]. In management science, it has been used to help theorize about how organizations adapt to solve complex problems [28] and to explain why it is often difficult for new firms to imitate successful incumbents [29]. In political science, the model has been adapted to explain why different political institutions perform better than others [30] and why diverse teams outperform homogeneous teams when solving complex problems [31].

The modeling framework abstracts key aspects of public health implementation decision-making, representing them mathematically so that the dynamics driving change over time can be explored. A program is defined as a sequence of decisions, represented by the vector *D*. For simplicity, each decision has only two options—yes (1) or no (0). For example, if there are five decisions in a sequence, a program could be represented by the bitstring [0,1,1,0,1]. Some programs are more effective than others, which is represented in the model by a *value function* *f*(*D*). The central challenge facing implementation is that this function is unknown; we wish to find the best program, but do not know in advance which set of decisions yields the highest value. The function *f*(·) is also called the *landscape*, and it is defined by three parameters, each of which corresponds to one of the three complexities described above.

### Landscapes

#### Dimensionality (*N*)

This parameter refers to the number of decisions that must be made when implementing a program. In vector notation, the program *D* is an ordered list of *N* component decisions, as follows:

$$D = [D_{1},\ldots,D_{N}] $$

Low dimensional problems are straightforward to solve through brute force. Each combination can be tried (perhaps using a factorial RCT), and the best program determined. Higher-dimensional programs, however, present a combinatorial challenge. If there are *N* decision to make, each with 2 options, then the total number of possible programs is equal to 2^*N*^, an exponential function. Table 1 illustrates the rapid growth of this function. When *N*=40, there are over a trillion possible programs, more than ten times the number of stars in the galaxy. In the face of this (literally) astronomical problem, there is simply no way to conduct an exhaustive evaluation of each possible program. Instead, any implementation procedure must accept some uncertainty and rely on heuristics to draw inferences from a limited set of program evaluations^1^.

**Table 1:** The exponential growth of programs

**Decisions**	**Programs**
1	2
2	4
5	32
10	1024
20	1,048,576
30	1,073,741,824
40	1,099,511,627,776

#### Ruggedness (*K*)

**Fig. 1:**
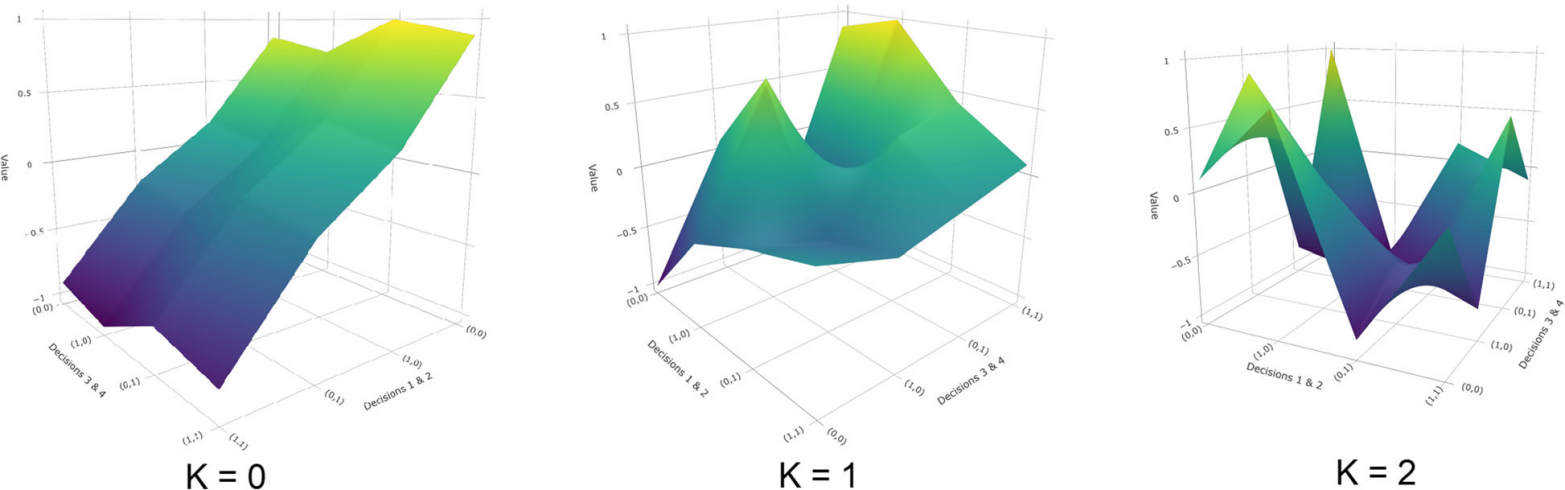
Illustrative landscapes. The number of local peaks in the fitness landscape increases with *K* (*N*=4 above)

This parameter is termed *ruggedness*, because it controls the smoothness or ruggedness of the fitness function. Whereas *N* describes the number of decisions that make up a program, *K* describes how those decisions interact to create value. When *K*=0, the value function *f*(·) is purely additive. The value of each decision does not depend on any other decisions; we simply add up the value of each decision to compute the total value of the program. Rugged problems (*K*>0), on the other hand, have multiplicative value functions. The value of each decision depends on the choice made at *K* other decision points. Decisions cannot be made independently, as the value of one choice depends on several other choices made. Figure 1 illustrates this transformation; as the value of *K* increases, the value function becomes more rugged, with multiple peaks and valleys dotting the landscape. This ruggedness increases the difficulty of the search problem. When *K* is small, the value function is smooth, and it is relatively straightforward to find the global optimum through incremental adjustment. But for large values of *K*, adjacent locations may have very different fitness values, and there is no guarantee that incremental adjustments to programs will eventually yield the highest-value program. Instead, one is more likely to become “stuck” on a local peak.

#### Context-specificity (*S*)

To reflect the fact that the effectiveness of programs may vary based on context, we introduce a third parameter called *S*. This parameter allows the value function to vary by context, so that each agent is attempting to solve a unique but correlated problem. Formally, the value of a program is equal to the weighted average of two functions, as follows:

$$f(D)={Sf}_{L}(D)+(1-S)f_{G}(D) $$

The function *f*_*G*_ is a global value function, shared in common across contexts, while *f*_*L*_ is a local value function, unique to each context. The parameter *S* is bounded between 0 and 1, so that *f*(·) is a convex combination of the two functions. When *S*=0, problems are *universal*; solutions discovered by one agent will work equally well for all agents. As *S* increases, so does the weight on the idiosyncratic *f*_*L*_ function. When *S*=1, problems are perfectly context-specific; each agent has an uncorrelated value function to solve. In this regime, there is little that can be learned across contexts; programs that perform well for one agent are no more likely than chance to perform well for another.

### Agents

The agents in our model represent public health practitioners (perhaps state public health agencies) attempting to implement a program that maximizes the value of the landscape function *f*(·). The agent-based model allows us to explore how several implementation procedures perform as we vary the key parameters of the landscape function, *N*, *K*, and *S*. The model unfolds over a series of *time steps*, and during each step, agents employ one of following three procedures to search the landscape for effective programs. These procedures are iterative (i.e., they are repeated multiple times over the course of the simulation), and the following sections describe the actions that agents take during a single time step.

#### Plan-Do-Study-Act

The simplest search procedure is a “hill climbing” or “random mutation” algorithm, analogous to a Plan-Do-Study-Act (PDSA) loop from the quality improvement literature [32, 33]. The PDSA loop—originally developed in the context of quality control in manufacturing—is an iterative process that emphasizes repeated testing and modification of one’s plans in response to evidence. In the agent-based model, agents employing this procedure do so as follows. First, create a new program *D*^′^ by changing the value of one decision point in *D*. Next, they evaluate this incrementally altered program. If *f*(*D*^′^)>*f*(*D*), they continue to implement *D*^′^. Otherwise, they revert to the original program *D*. This incremental quality improvement procedure can be repeated as many times as necessary.

#### Evidence-based interventions

When agents in the model employ evidence-based interventions (EBIs), they adopt programs that have proven effective for other agents. If PDSA is analogous to random mutation, then EBI is analogous to evolutionary selection, in which the “fittest” programs are replicated by other agents. In the model, EBI is represented by an agent discontinuing their current program, and instead implementing the highest-value program discovered by another agent in the simulation. Note that in the results presented below, agents employ a mixture of both PDSA and EBI so that they do not all converge on the same program instantaneously.

#### Evidence-based decision-making

We can think of evidence-based interventions as a sort of asexual selection strategy. Successful designs create identical copies of themselves whenever agents imitate the “fittest” strategies found by other agents. To push the evolutionary biology analogy one step further, we could instead imagine an alternative search procedure akin to sexual selection. Agents using this procedure, which we will call evidence-based decision-making (EBDM), construct programs by imitating multiple “parents.” Rather than wholly adopting a single, high-value program from another context, agents using EBDM recombine the constituent decisions from several high-value programs, adopting piecewise the decisions that are common across them. For example, suppose that the following two programs— *D*_*A*_ and *D*_*B*_—were evaluated and discovered to have a high value.

$$D_{A}=[0,1,1,0,1,0]\\ D_{B}=[0,0,1,0,0,1] $$

An agent combining these programs with EBDM would adopt all of the decisions that the two programs share in common (in this case, decisions 1, 3, and 4). For the decisions where the two programs disagree, the agent would not alter its current program. Such a process may, for example, yield the following program *D*^′^:

$$D'=[0,1,1,0,1,1] $$

### Model dynamics

Agents begin the simulation with a random program. Each time period, agents search for effective programs using one of the three implementation strategies. To reflect the time and resource constraints under which real-world public health agencies operate, we only allow agents to search the landscape for 100 time periods. We then rerun the simulation 50 times at each parameter combination to account for stochasticity, computing summary statistics across runs. See the Technical Appendix for more information on the computational implementation of the model, pseudocode, and replication materials.

## Results

For ease of presentation, we report the model’s results in two parts, first focusing on universal problems (i.e., landscapes with *S*=0) then context-specific problems (i.e., landscapes with *S*>0).

### Universal problems

**Fig. 2:**
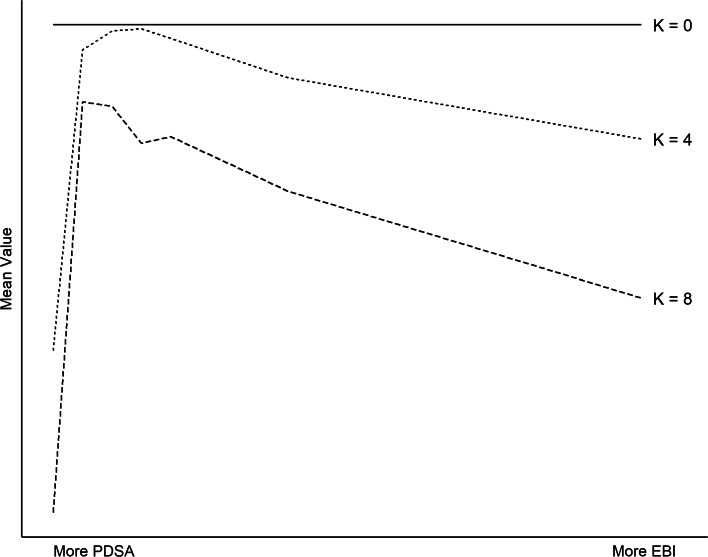
Universal problems. When *K* is low, PDSA alone performs well. As *K* increases, a mixture of PDSA and EBI performs best. (Parameters used to generate figure: *N*=10 and *S*=0.)

To begin, consider only landscapes with *S*=0 (no context-specificity). These are *universal*, where the effectiveness of an intervention does not depend on context (e.g., clean indoor air policies to reduce secondhand smoke exposure [34]). Figure 2 plots the mean value of interventions implemented by agents at the end of each simulation, varying *K* and the mixture of implementation strategies employed. As the solid line illustrates, when *K*=0 (smooth landscape), PDSA alone can reliably find the highest-value intervention, regardless of dimensionality (*N*) or context-specificity (*S*). To see why, recall that when *K*=0, the value function *f*(·) is strictly additive; the value of each decision does not depend on the choices made elsewhere. This means that each decision can be evaluated independently. If changing one decision yields a higher value, then that is the optimal choice. In this manner, the highest-value program can be discovered in no more than *N* steps.

For higher values of *K* (rugged landscapes), PDSA alone is a less useful strategy. Intuitively, this is because a pure hill climbing procedure in rugged terrain is likely to yield a *local* peak (the highest-value program in a neighborhood) rather than a *global* peak (the highest-value program overall). To illustrate, consider Fig. 1. In the leftmost panel (*K*=0), there is a single peak, which can be reach by hill climbing in no more than 4 steps. In the rightmost panel (*K*=2), there are six local peaks. Depending on where one begins, PDSA is most likely to yield one of these local peaks rather than the global best program. When agents in our computational model pursue PDSA alone, every agent finds the best possible program when *K*=0. As *K* increases, the percentage of agents that implement the best possible program declines, and in the limit (*K*=*N*−1), nearly every agent fails to implement the global peak.

On such rugged landscapes, agents that adopt EBI perform significantly better than those that ignore evidence from other contexts. However, a surprising result from the model is that too much EBI can undermine effectiveness as well. Indeed, mean value peaks when agents employ a *mixture* of EBI and PDSA. This finding is analogous to what we observe in evolutionary biology, where a combination of mutation and selection outperforms either procedure alone. Intuitively, EBI acts as a sort of parallel processing approach to problem-solving. Rather than a single agent searching the space for a good solution, multiple agents search simultaneously, and over time, they adopt the most successful programs found by the group. But too much EBI can be counterproductive, particularly on rugged landscapes (high *K*). When agents adopt EBI without performing their own quality improvement, they are more likely to converge on a local peak than the global optimum. This result highlights the importance of not sacrificing local knowledge entirely in favor of outside solutions.

### Context-specific problems

**Fig. 3:**
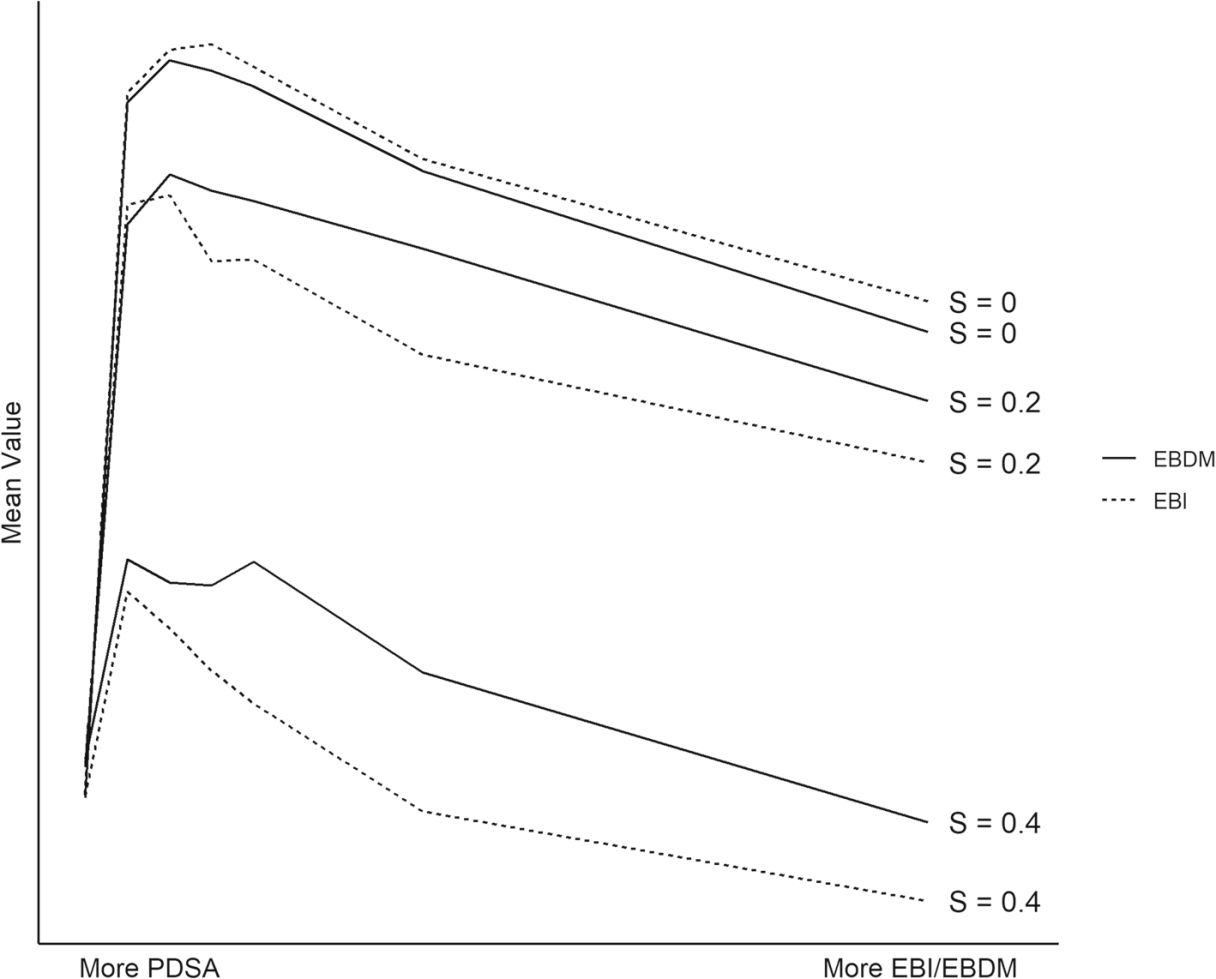
Context-specific problems. When *S*>0, a combination of EBDM and PDSA outperforms all other implementation procedures. (Parameters used to generate figure: *N*=10 and *K*=4.)

Next, consider landscapes with *S*>0. For these sorts of *context-specific* problems, a single program that works well for one agent is unlikely to produce similar results in another context (e.g., a folic acid mass market campaign that may have different effectiveness depending on SES [34]). But because the landscapes are correlated, individual *decisions* that yield high value across multiple contexts are likely to be good choices. As a result, evidence-based decision-making (EBDM) tends to outperform pure EBI. Figure 3 illustrates this result, varying the value of *S* and the mixture of implementation procedures employed (PDSA on the left versus EBDM/EBI on the right). In simulations where *S* is large, EBDM yields consistently higher value programs than EBI alone, regardless of the value of *N* or *K*.

The advantage of the EBDM approach is twofold. First, it preserves the diversity of programs being pursued within a population. When pursuing pure EBI—adopting the single highest performing program—agents quickly converge a single set of decisions. Such homogeneity is problematic when solving complex problems, as a large number of agents implementing identical programs may fail to sufficiently explore the space and discover good solutions. This is especially true when *S* is large and there does not exist an optimal one-size-fits-all program. But evidence-based decision-making does not suffer from this defect; just as in evolutionary biology, “children” are a mix of their parents’ genes rather than identical copies. This preserves the diversity of programs, improving the ability of the system as a whole to find better solutions.

Second, the EBDM approach leverages more information than EBI alone. As discussed above, we can conceptualize EBI is as a form of parallel computation, with multiple agents solving a problem simultaneously and communicating their results. In this regard, EBDM is an improved form of parallel processing, where agents learn from more than one other agent at a time. Over the course of the search, multiple agents may discover high-value programs that have somewhat different component decisions. Taking what works from these programs can yield higher value than replicating any single program alone. (Note, however, that when *S*=0, EBDM performs strictly worse than EBI in our simulations. When one is certain that a problem is truly universal and that good programs will transfer across context, then there is no longer an advantage to EBDM.)

## Discussion

The model and results presented here have four significant implications for our understanding of how and why mis-implementation occurs in public health practice.

First, the model demonstrates the importance of evaluating the results of interventions and carefully documenting the decisions made. Without such evaluation and detailed communication, there is no way to leverage the parallel computation engine that performs best at quickly finding good solutions. For all but the simplest problems, PDSA alone is unlikely to yield effective programs. Communicating evidence across agencies is essential for tackling complex problems, and the development of reporting guidelines and checklists [35] is a promising step in the right direction.

Second, the results caution against an approach to EBI that is overly proscriptive. If evidence-based interventions are adopted too rapidly and uncritically in the face of complex problems and varied contexts, results are likely to be poorer. Instead, the best results obtain when agents are able to combine local knowledge with evidence-based decisions from other contexts. One example of this approach to public health practice in the USA is the Centers for Disease Control and Prevention’s Preventive Health and Health Services Block Grant Program, which provides funding to state, tribal, and territorial agencies in a flexible manner. Through this funding, agencies are provided funding to address public health needs unique to their populations with community-driven methods and evidence-based interventions. There are general guidelines in terms of the domains of programming (e.g., preventive screenings and services, environmental health), but the agencies have autonomy over how exactly to spend the Block Grant funding and what specific EBIs to implement.

Third, the model lends additional support to EBDM. In a related setting (mental health), Massatti and colleagues made several key points regarding mis-implementation: (1) the right mix of contextual factors (e.g., organizational support) is needed for continuation of effective programs in real-world settings, (2) there is a significant cost burden of the programs to the agency, and (3) understanding the nuances of early adopters promotes efficient dissemination of effective interventions [36]. Management support for EBDM in public health agencies is associated with improved public health performance [37, 38]. In a cross-country comparison of mis-implementation, leadership support and political contexts were all common factors in whether programs continued or ended [2]. Preliminary data indicate that organizational supports for EBDM may be protective against mis-implementation (e.g., leadership support for EBDM, having a work unit with the necessary EBDM skills) and, more specifically, a leader’s ability to persevere in implementation of EBIs, ability and willingness to manage change, and use of quality improvement processes [1, 3].

Finally, the model suggests that the context in which implementation decisions are made can be deeply important for crafting effective programs. Public health challenges and health department settings vary widely across the USA, and for many types of problems, it may be unwise to apply a one-size-fits-all approach to decision-making. Policymakers should carefully consider whether the problem they are facing is universal (*S*=0) or context-specific (*S*>0), and if it is the latter, adopt the sorts of evidence-based decision-making procedures described here. Models such as ours present a useful tool for understanding how decision-making around program implementation occurs across diverse contexts.

## Conclusions

To our knowledge, this is the first application of rugged landscape theory to public health and implementation science. Although the model is highly abstract, it reveals counterintuitive dynamics that can help explain the puzzling persistence of mis-implementation in practice. These results suggest promising avenues for future work focused on reducing mis-implementation and enhancing population health, and in forthcoming research, we plan to expand on this model to incorporate additional factors that influence implementation success, including the internal dynamics of decision-making within public health departments.

## Supplementary information

**Additional file 1** Technical Appendix. Contains technical information on the model’s implementation, pseudocode, and links to replication materials.

## Data Availability

All datasets and replication code are available from the corresponding author on reasonable request.

## Abbreviations

ABM: Agent-based modeling
EBI: Evidence-based intervention
EBDM: Evidence-based decision-making
NKS: N = dimensionality, K = ruggedness, S = context-specificity
PDSA: Plan-Do-Study-Act
RCT: Randomized control trial

## Footnotes

1 Computer scientists refer to such problems as “NP-Complete.” There is no known method that guarantees finding the best solution in polynomial time.
