# Supplementary material for: Rugged landscapes: complexity and implementation science
Source: Implement Sci. 2020 Sep 29;15:85. doi: 10.1186/s13012-020-01028-5 (PMC7523395; doi:10.1186/s13012-020-01028-5)
Supplement: Supplementary file 1 — Additional file 1 Technical Appendix. Contains technical information on the model’s implementation, pseudocode, and links to replication materials. [file 13012_2020_1028_MOESM1_ESM.pdf]

Technical Appendix for "Rugged Landscapes: Complexity and Implementation Science"

Joseph T. Ornstein

March 10, 2020

R Code

The following `R` code reproduces the results from the paper.

Define Parameters

First, define the three complexity parameters.

```
N <- 10
K <- 6
S <- 0.6
```

Create The Fitness Landscape

The fitness landcape is the weighted sum of `fitness_global` and `fitness_local`, weighted by the parameter `S`. See the "Landscape Functions" section for complete definitions of the auxiliary functions called here.

```
policySpace <- generatePolicySpace(N)

fitness_global <- generateFitnessLandscape(N, K)
fitness_local <- generateFitnessLandscape(N, K)

fitness <- weightedLandscape(fitness_global, fitness_local, S)
```

Agent-Based Model (ABM)

```
# Number of iterations per parameter combination
numIterations <- 50

# Dataframe for output
dat <- expand.grid(N = 10,
                   K = c(0,2,4,6,8),
                   S = c(0.6, 0.7, 0.8, 0.9, 1),
                   run = 1:numIterations,
                   p = c(0, 0.025, 0.05, 0.075, 0.1, 0.2, 0.5),
                   misimplementation.rate.75.EBI = NA,
                   misimplementation.rate.75.EBD2 = NA,
                   misimplementation.rate.75.EBD3 = NA,
                   mean.value.EBI = NA,
                   mean.value.EBD2 = NA,
                   mean.value.EBD3 = NA,
                   sd.value.EBI = NA,
                   sd.value.EBD2 = NA,
                   sd.value.EBD3 = NA)
```

```r
# Core Loop
for (i in 1:nrow(dat)){

  policySpace <- generatePolicySpace(dat$N[i])
  fitness_global <- generateFitnessLandscape(dat$N[i],dat$K[i])

  EBI.values <- hillClimbwithEBI(num.agents = 50, num.iterations = 100,
                                 q = 1, EBI.weight = dat$p[i],
                                 N = dat$N[i], K = dat$K[i], S = dat$S[i])
  EBD2.values <- hillClimbwithEBD(num.agents = 50, num.iterations = 100,
                                 q = 1, EBD.weight = dat$p[i], m = 2,
                                 N = dat$N[i], K = dat$K[i], S = dat$S[i])
  EBD3.values <- hillClimbwithEBD(num.agents = 50, num.iterations = 100,
                                  q = 1, EBD.weight = dat$p[i], m = 3,
                                  N = dat$N[i], K = dat$K[i], S = dat$S[i])

  dat$mean.value.EBI[i] <- mean(EBI.values)
  dat$mean.value.EBD2[i] <- mean(EBD2.values)
  dat$mean.value.EBD3[i] <- mean(EBD3.values)

  dat$sd.value.EBI[i] <- sd(EBI.values)
  dat$sd.value.EBD2[i] <- sd(EBD2.values)
  dat$sd.value.EBD3[i] <- sd(EBD3.values)
}
```

Landscape Functions

These functions generate the fitness landscapes.

```r
# Generate the set of programs: all combinations of decisions
generatePolicySpace <- function(N){
  policies <- matrix(rep(c(0,1),N),nrow = 2) %>%
    as.tibble

  policySpace <- expand.grid(policies)

  return(policySpace)
}

# Return a random interaction matrix
imatrix <- function(N,K){

  toReturn <- matrix(0,nrow = N, ncol = N)

  for (i in 1:N){
    #Select K+1 indices, including self
    indices <- c(sample(1:N) %>% setdiff(i), i)[(N-K):N]
    for (j in sort(indices)){
      # we turn on those interactions
      toReturn[i, j] <- 1
    }
  }

  return(toReturn)
```

```
}


# Assign fitness values to each program
generateFitnessLandscape <- function(N, K){

  #Create a random interaction matrix
  interaction_matrix <- imatrix(N,K)

  #Start with a 2^N by N table of uniform[0,1] random numbers
  NK_land <- matrix(runif(2^N * N, 0, 1), nrow = 2^N)

  #Need this sequence (the "power key") to associate {0,1}^N with a row in the dataframe
  power_key <- 2^(0:(N-1))

  # Calculate fit vector (helper function to generate fitness landscapes)
  calculateFit <- function(decision){

    fit_vector <- rep(0,N)
    for (i in 1:N){
      fit_vector[i] <- NK_land[sum(decision * interaction_matrix[i,] * power_key) + 1, i]
    }

    return(fit_vector)
  }

  fitness_contributions <- apply(policySpace, 1, calculateFit) %>% t

  #Then fitness is the mean of each row
  fitness <- rowMeans(fitness_contributions)

  #Normalize to [-1,1]: (x - min) / (max-min) - 0.5 * 2
  minfit <- min(fitness)
  maxfit <- max(fitness)
  fitness %<>% subtract(minfit) %>%
    divide_by(maxfit - minfit) %>%
    subtract(0.5) %>%
    multiply_by(2)

  return(fitness)
}

# Take the weighted average of two fitness landscapes (weighted by S)
weightedLandscape <- function(fitness_global, fitness_local, S){
  fitness <- S * fitness_local + (1-S) * fitness_global
  minfit <- min(fitness)
  maxfit <- max(fitness)
  fitness %>%
    subtract(minfit) %>%
    divide_by(maxfit - minfit) %>%
    subtract(0.5) %>%
    multiply_by(2) %>%
    return
```

```r
}

# Return the value of a program
getValue <- function(program, fitness){
  N <- length(program)
  power_key <- 2^(0:(N-1))
  return(fitness[sum(program * power_key) + 1])
}
```

Search Algorithms

These algoritms define the search procedures employed by agents in the model.

PDSA

```r
# Local Search with sophistication parameter
# (q = number of decisions an agent can alter at once)
hillClimb <- function(program, fitness, num_iterations, q){

  N <- length(program)
  value <- getValue(program, fitness)
  bestValue <- max(fitness)

  iterations <- 0
  while (iterations < num_iterations){

    #Modify the program (change q bits)
    new_program <- program
    bits <- sample(1:N, q, replace = F)
    new_program[bits] <- abs(new_program[bits] - 1)

    new_value <- getValue(new_program, fitness)

    if(new_value > value){
      program <- new_program
      value <- new_value
    }

    iterations <- iterations + 1
  }

  return(program)

}
```

EBI

```r
# Get values for a set of programs and fitness landscapes
getValues <- function(programs, fitnessMatrix){
  values <- rep(0, nrow(programs))
  for (i in 1:nrow(programs)){
    values[i] <- getValue(programs[i,], fitnessMatrix[,i])
  }
```

```r
  return(values)
}

# From a set of programs, find the best one and adopt it.
getEBI <- function(programs, fitnessMatrix){
  values <- getValues(programs, fitnessMatrix)
  bestProgram <- programs[which.max(values),]
  return(list(bestProgram, max(values)))
}

hillClimbwithEBI <- function(num.agents, num.iterations, q, EBI.weight, N, K, S) {

  #Generate initial programs
  programs <- matrix(0, ncol = N, nrow = num.agents)
  for(i in 1:num.agents){
    programs[i,] <- policySpace[sample(1:nrow(policySpace),1),] %>% unlist
  }
  #Generate Fitness Landscapes
  fitnessLandscapes <- matrix(0, nrow = length(fitness), ncol = num.agents)
  for(i in 1:num.agents){
    fitnessLandscapes[,i] <- weightedLandscape(fitness_global,
                                               generateFitnessLandscape(N, K), S)
  }

  iteration <- 0
  while (iteration < num.iterations){

    #Let each agency take a turn
    for (i in 1:nrow(programs)){

      # With probability = EBI.weight, adopt the best program.
      # Otherwise, conduct one local search.
      if (runif(1,0,1) < EBI.weight) {
        programs[i,] <- getEBI(programs, fitnessLandscapes)[[1]]
      } else {
        programs[i,] <- hillClimb(program = programs[i,],
                                  fitness = fitnessLandscapes[,i],
                                  num_iterations = 1, q = 1)
      }

    }

    iteration <- iteration + 1
  }

  values <- getValues(programs, fitnessLandscapes)

  return(values)
}
```

EBDM

```r
# Get the best m programs, adopt the majority vote on each decision.
getEBD <- function(programs, fitnessMatrix, m){
  values <- getValues(programs, fitnessMatrix)
  # get the best m programs.
  bestPrograms <- programs[order(values, decreasing=TRUE)[1:m],] %>% as.matrix
  if(m == 1) bestPrograms %<>% t

  # Take the majority vote from that vector
  # (round mean to nearest 0-1, if p=0.5, select at random)
  EBD <- colMeans(bestPrograms)
  for(i in 1:length(EBD)){
    if(EBD[i] == 0.5){
      EBD[i] <- sample(c(0,1),1)
    } else{
      EBD[i] <- round(EBD[i])
    }
  }
  return(EBD)
}

hillClimbwithEBD <- function(num.agents, num.iterations, q, EBD.weight, m, N, K, S) {

  #Generate initial programs
  programs <- matrix(0, ncol = N, nrow = num.agents)
  for(i in 1:num.agents){
    programs[i,] <- policySpace[sample(1:nrow(policySpace),1),] %>% unlist
  }
  #Generate Fitness Landscapes
  fitnessLandscapes <- matrix(0, nrow = length(fitness_global), ncol = num.agents)
  for(i in 1:num.agents){
    fitnessLandscapes[,i] <- weightedLandscape(fitness_global,
                                               generateFitnessLandscape(N, K), S)
  }

  iteration <- 0
  while (iteration < num.iterations){

    #Let each agency take a turn
    for (i in 1:nrow(programs)){

      #With probability = EBD.weight, get the EBD. Otherwise, conduct one local search.
      if (runif(1,0,1) < EBD.weight) {
        programs[i,] <- getEBD(programs, fitnessLandscapes, m)
      } else {
        programs[i,] <- hillClimb(program = programs[i,],
                                  fitness = fitnessLandscapes[,i],
                                  num_iterations = 1, q = 1)
      }

    }

    iteration <- iteration + 1
  }
```

```r
  values <- getValues(programs, fitnessLandscapes)

  return(values)
}
```
